# Supplementary material for: Physiological and stem cell compartmentalization within the Drosophila midgut
Source: eLife. 2013 Aug 27;2:e00886. doi: 10.7554/eLife.00886 (PMC3755342; doi:10.7554/eLife.00886)
Supplement: Supplementary file 1. — (A) RNASeq analysis of Drosophila midgut. (B) Regional expression of midgut genes. DOI: http://dx.doi.org/10.7554/eLife.00886.013 [file elife00886s001.docx]

**Supplementary file 1A. RNASeq analysis of Drosophila midgut**

| region^1^ | line^2^ | reads^3^ | total reads | genes^4^ |
| --- | --- | --- | --- | --- |
| A1 | 50A12,46B08 | 33.6, 44.0, 36.7 | 114 | 9,380 |
| A2-A3 | 50A12 | 38.7, 51.1, 55.3 | 145 | 9,251 |
| Cu | 50A12 | 105.3, 36.7, 58.2 | 200 | 10,031 |
| LFC, Fe | 50A12 | 30.9, 34.6, 64.5 | 130 | 9,509 |
| Fe | 46B08 | 27.2, 93.0, 90.0 | 210 | 9,266 |
| P1 | 46B08 | 56.9, 68.4, 92.5 | 218 | 9,653 |
| P2-P4 | 46B08 | 69.1, 54.4, 56.3 | 180 | 9,695 |
| A1-A3 | 42G03 | 43.1, 31.7, 57.7 | 132 | 9,740 |
| Cu, LFC, Fe | 46B08,42G03 | 46.8, 70.3, 62.1 | 179 | 9,604 |
| P1-P4 | 46B08,42G03 | 109.5, 67.6, 37.5 | 214 | 9,928 |

^1^name of midgut subregion, see Figure 2B

^2^the Janelia Farm GAL4 line used with UAS-GFP to isolate the region

^3^reads (in millions) i.e. reads x 10E-06

^4^number of genes with fpkm >0.05 and S.D./mean <1.

**Supplementary file 1B. Regional expression of midgut genes**

| subregion^1^ | A1 | A23 | Cu | LFC/Fe | Fe | P1 | P234 | A123 | CuLFCFe | P1234 |
| --- | --- | --- | --- | --- | --- | --- | --- | --- | --- | --- |
| A1^2^ | X | **87** | **250** | **177** | **214** | **218** | **188** | X | X | X |
| A23^2^ | **60** | X | **210** | **108** | **142** | **117** | **123** | X | X | X |
| Cu^2^ | **168** | **135** | X | **64** | **95** | **181** | **149** | X | X | X |
| LFC/Fe^2^ | **143** | **120** | **165** | X | **34** | **83** | **120** | X | X | X |
| Fe^2^ | 137 | 107 | 150 | 32 | X | 72 | 13032 | X | X | X |
| P1^2^ | 201 | 277 | 280 | 107 | 177 | X | 60 | X | X | X |
| P234^2^ | 295 | 232 | 361 | 201 | 266 | 124 | X | X | X | X |
| A123^3^ | X | X | X | X | X | X | X | X | **303** | **263** |
| CuLFCFe^3^ | X | X | X | X | X | X | X | **437** | X | **259** |
| P1234^3^ | X | X | X | X | X | X | X | **474** | **356** | X |

^1^name of midgut subregion, see Figure 2B

^2^number of genes with fpkm>1, SD/mean <1, and >10X higher fpkm than the indicated region

^3^number of genes with fpkm>0.2, SD/mean <1, and >5X higher fpkm than the indicated region
